# Tough times for seasoned equity offerings: performance during the COVID pandemic

**DOI:** 10.1007/s11573-022-01089-6

**Published:** 2022-04-13

**Authors:** Marc Zenzius, Christian Flore, Dirk Schiereck

**Affiliations:** Department of Business Administration, Economics and Law, Technische Universität Darmstadt, Hochschulstr. 1, 64289 Darmstadt, Germany

**Keywords:** Covid, SEO, Event study, G14

## Abstract

This study analyzes the wealth effects of SEO announcements in the US during the COVID-19 pandemic and its main determinants. We find significantly negative abnormal returns of − 8.6%. This provides persuasive evidence that capital markets reacted particularly negative during this period, reflecting higher degrees of uncertainty. We furthermore find that larger firms experience a better SEO performance and that COVID-19 related biotech & healthcare firms react particularly negative. This effect is more negative the lower the company valuation beforehand.

## Introduction

At the end of 2019, the SARS-CoV-2 virus and the COVID-19 disease it caused spread across China for the first time. In the beginning of 2020 this development reached the rest of the world also severely affecting Europe and the United States. The WHO declared the disease to be a pandemic on the 11th of March, when COVID-19 was already spreading exponentially in the U.S. (Centers for Disease Control and Prevention 2021; WHO 2020). Measures to curb the pandemic’s dynamic were unprecedented in peace time and stock markets reflected the global grip of the pandemic (e.g., Park et al. 2008; Schiereck et al. 2016).

These are tough times for corporations, with sales markets breaking in and business operations being restricted. Stock markets have been very volatile since the pandemic’s inception with persistent high degrees of uncertainty, arguably making it harder for companies to obtain equity financing. With this paper we address the question on how markets value the announcement of a firm’s seasoned equity offering (SEO) during these extraordinary times. To this end, we study the stock price reactions to SEO announcements in the United States. Further, we examine whether the announcement effect’s heterogeneity can be explained by a firm’s affiliation to a certain industry. This is a natural question as industries are affected quite differently by the pandemic and the lockdown measures taken to control its course. We are particularly interested in the most attractive industry segment during the pandemic, namely the COVID-19 related biotech and healthcare industry. These companies are affected quite differently by the pandemic than companies from other industries in the sense that the pandemic provides earnings growth opportunities for these firms while it is generally bad news for other firms. For this, we examine a representative sample of 297 SEO issues announced in the US between 11.03.2020—the date the WHO declared COVID-19 to be a pandemic—and 30.09.2020.

We contribute to the existing literature by studying the effect of industry affiliation on SEO announcement effects in the context of the COVID pandemic. Different industries face diverse challenges and chances during the pandemic. We thereby explore the mechanism of the SEO announcement effect during a crisis. We find that companies that profit from the crisis incur a more negative announcement effect, which may indicate higher degrees of information asymmetry. Former studies have tried to capture and explain the announcement effect in general (Masulis and Korwar 1986; Mikkelson and Partch 1986; Myers and Majluf 1984). Recent studies have focused on more specific questions like on offer date revisions, customer supplier relationships, or weekday of the announcement (Chan et al. 2018; Johnson et al. 2018; Michaely et al. 2016). This paper examines the relevance of individual stock run-ups (Masulis and Korwar 1986), firm valuation (Jung et al. 1996), firm size (Guo and Mech 2000), profitability (Mueller 1972; Myers and Majluf 1984) and deal size (Intintoli and Kahle 2010). We thereby find that only firm size yields significant explanatory power during the COVID-19 pandemic.

Furthermore, we add to the existing literature on the COVID-19 pandemic’s impact on the economy by examining the impact on equity valuations and SEO announcement effects. So far, studies have mostly focused on the pandemic’s general impact on financial market performance and volatility (Baig et al. 2020; Baker et al. 2020; Zaremba et al. 2020; Zhang et al. 2020).

The remainder of the paper is structured as follows. Section 2 highlights some characteristics that distinguish COVID from non-COVID times. Section 3 gives a review of relevant literature and the underlying theories. Section 4 introduces the applied methodology and describes the underlying sample. Subsequently, Sect. 5 contains the empirical results of the event study and Sect. 6 analyzes the driving factors of the price reaction. This study closes with Sect. 7, the conclusion and a summary of the findings.

## COVID vs. non-COVID times

Before we analyze the impact of the COVID pandemic on SEO announcement effects we look at its effect on various economic indicators and we compare the pandemic to the economic consequences of the global financial crisis (GFC) of 2007/08.

**Fig. 1:**
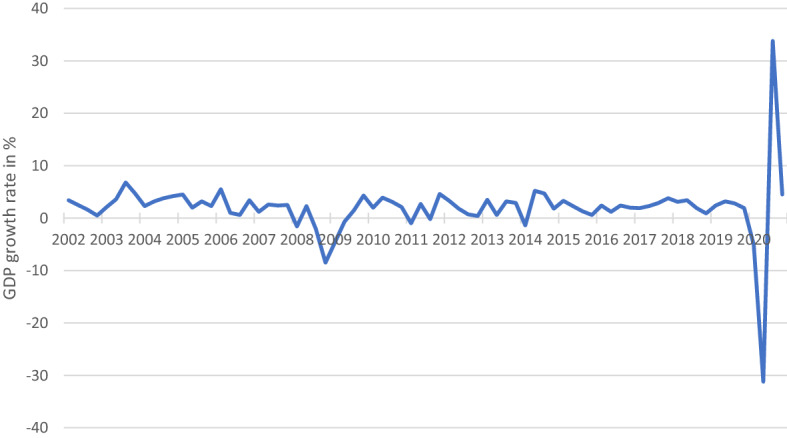
U.S. real quarterly GDP growth rate in percent from 2002 to 2020

Figure 1 shows the GDP growth of the United States in real terms on a quarterly basis from 2002 until the end of 2020 (Bureau of Economic Analysis 2020). The figure underlines the massive contraction of the US Economy in the beginning of the pandemic which is distinctly larger than the downturn during the GFC. Following this initial contraction, the subsequent recovery then occurs much quicker than after the GFC again with much larger growth rates.

**Fig. 2:**
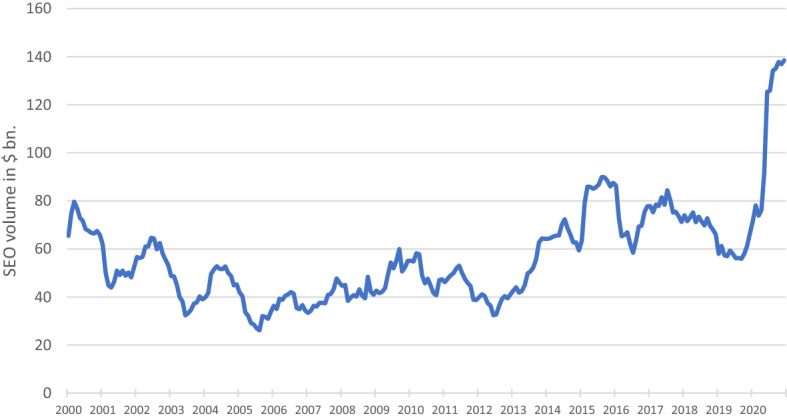
SEO volume of non-financial firms as a 12-months moving sum in $ bn. from 2000 to 2020

Next, we consider the SEO volume during the pandemic and also during pre-pandemic times. Figure 2 presents the SEO activity of non-financial firms as a 12-months moving sum (Board of Governors of the Federal Reserve System (U.S.) 2020). It shows that SEO activity increases significantly in 2020 and reaches its 20 years high as early as in May indicating extraordinary volumes of SEOs during the pandemic.

**Fig. 3:**
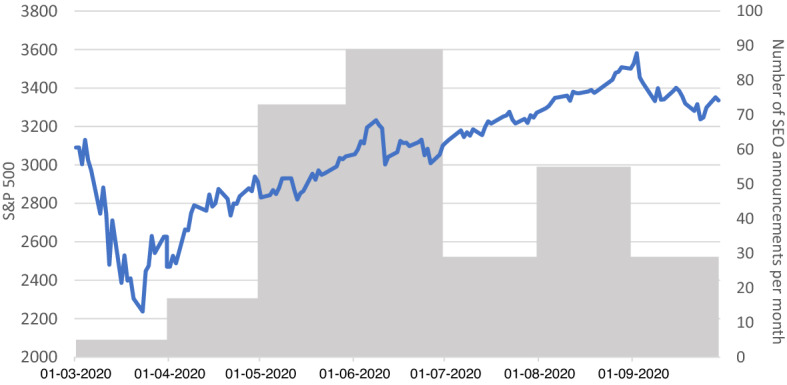
Number of SEO announcements per month vs. S&P 500 over the investigation period. The development of the S&P 500 index is depicted as a blue line and is measured on the left vertical axis. The grey bars measure the number of SEO announcements per month and correspond to the right vertical axis

Lastly, we consider the development of the S&P 500 compared to the number of SEO announcements in our sample as shown in Fig. 3. As the market contracts during March, the number of SEOs is very low. During the following recovery the number of SEO announcements increases massively, peaking in May and June of 2020.

These economic indicators underline the COVID pandemic’s significant impact on economic activity. An initial massive economic downturn is followed by a quick and also massive economic recovery. The SEO volumes thereby are on unprecedent high levels during the pandemic. These circumstances are quite different to the global financial crisis, where downturn and recovery took place during a more extended time frame and SEO activity was not at extraordinarily high levels. These circumstances underline the peculiar setting the COVID pandemic poses for research, specifically into SEO activity.

## Review of literature and hypothesis development

### Related literature

The majority of research on the topic of SEOs is based on the equity market in the United States. These studies consistently find significant negative price reactions in the form of abnormal returns (Veld et al. 2018). A number of studies is also concerned with price reactions of capital markets outside the U.S. (e.g., Bøhren et al. 1997; Iqbal 2008).

Capital market reactions to SEOs can be generally explained by the signaling theory which states that “markets are characterized by informational differences between buyers and sellers” (Leland and Pyle 1977). First, following the implied cashflow hypothesis, the announcement of a SEO signals that the firm’s managers anticipate lower future cashflows (Miller and Rock 1985). Consequently, a firm’s leverage needs to be reduced in order to maximize its valuation (DeAngelo and Masulis 1980). Hence, a rational investor reacts negatively to a SEO announcement. This theory is supported by empirical evidence provided by Downes and Heinkel (1982) and Masulis and Korwar (1986).

A second explanation considers market timing and states that the management of a firm acts in favor of existing shareholders. Thus, managers, having superior information to the market, issue new equity only when the firm’s stock price is overvalued (Myers and Majluf 1984). Consequently, rational investors discount the stock price after a SEO announcement, as the firm’s management has signaled that the stock is overvalued. This theory is also related to the pecking order hypothesis as the deviation from the pecking order signals a possible overvaluation (Myers 1984). The impact of information asymmetry between a firm’s managers and potential stockholders on the announcement effect is supported empirically by numerous studies, such as those by Eckbo and Masulis (1995), Masulis and Korwar (1986) and Dierkens (1991) or more recently Demiralp et al. (2011).

Third, the agency cost theory focuses on the costs that arise from “the separation of ownership and control” (Jensen and Meckling 1976). Instead of acting on behalf of existing shareholders, managers seek to maximize their own utility. Thus, managers deviate from the pecking order hypothesis and finance negative NPV-projects which increase their utility with equity rather than debt (Jung et al. 1996). Consequently, the announcement of a SEO leads to negative price reactions. Jung et al. (1996) and Holderness (2018) provide substantial empirical support for the agency cost theory.

Empirical studies have documented negative price reactions to SEO announcements and examined the driving factors influencing these reactions (Veld et al. 2018). During periods of market expansion—i.e., “periods with more promising investment opportunities”—reactions to SEO announcements are less negative (Choe et al. 1993). It can be argued that uncertainty regarding the firm’s value and information asymmetry is less pronounced during expansionary periods. Accordingly, more firms issue equity during these favorable market conditions (Henderson et al. 2006). This also corresponds with findings that show that price reactions are two percentage points less severe in hot markets than in cold markets (Bayless and Chaplinsky 1996). Bayless and Chaplinsky differentiate regarding the timing of SEOs, introducing hot periods and cold periods. These findings are robust to the different macroeconomic characteristics of the hot and cold periods. Firms with a higher price-to-book ratio experience less pronounced price reactions upon SEO announcement. Dierkens (1991) argues that a high price-to-book ratio can be found with firms that have predominantly intangible assets and valuable projects.

Alike, firms with “the most valuable investment opportunities do not experience adverse stock returns when they issue equity” (Jung et al. 1996). This statement holds, more general than the findings regarding market expansions, independently from the markets state. This also supports findings that show a general positive correlation of price reaction and growth opportunities (Dierkens 1991).

There is evidence that the price run-up in the pre-announcement period is “used by the market in predicting stock offering announcements” (Masulis and Korwar 1986). As a price run-up lowers a firm’s leverage the market considers an upcoming SEO, which would further reduce the leverage, as less likely. This leads to an even stronger signaling effect and stronger market reactions upon SEO announcement.

The reaction to seasoned equity offerings is more negative for smaller relative issue sizes. As greater issues put the manager’s wealth at stake, it is suggested that they pressure underwriters to reduce underpricing (Intintoli and Kahle 2010). Asymmetric information tends to be lower for larger firms, as they attract more media attention and are followed more closely by financial analysts, reducing information asymmetry (Guo and Mech 2000). Therefore, price reactions on announcement days should be less pronounced for larger firms. The magnitude of asymmetric information decreases at a decreasing rate with the firm’s size. Harris and Raviv (1991) verify this statement and argue that the price drop is larger when there is a lower market capitalization and hence more information asymmetry.

Similarly, using the number of analysts to represent the degree of asymmetric information, firms with a smaller number of financial analysts incur more negative abnormal returns around the announcement day (D’Mello and Ferris 2000). This is also in line with Guo and Mech (2000).

Regarding the recent empirical research on the COVID-19 pandemic, stock markets in general reacted with consistent negative abnormal returns over a period of 20 days after the first case of a COVID-19 infection. This holds for 30 different markets (Baker et al. 2020). COVID-19 also effects the liquidity and volatility of financial markets (Baig et al. 2020). An increase in COVID-19 infections, COVID-19 related deaths and restrictive government policies impair market liquidity and lead to an increase in volatility (Zaremba et al. 2020). This resonates with the more general results of empirical research like that of Blau et al. (2014) which show that economic freedom and market volatility have an inverse relation. COVID-19 also leads to an increase in systematic market risk, with the magnitude on a national level being dependent on the severity of the outbreak in the specific country (Zhang et al. 2020).

### Hypothesis development

Based on existing literature, we develop two hypotheses. The first hypothesis concerns the market reaction to SEO announcements. Various studies have shown that SEO announcements in the U.S. trigger negative market reactions. Myers and Majluf (1984) argue that SEO announcements reduce information asymmetry between managers and the market and thereby signal unfavorable information to investors. Thus, equity issues are predicted to be associated with negative abnormal returns. To measure information asymmetry, former studies have used firm size (Guo and Mech 2000), economic conditions (Bayless and Chaplinsky 1996; Choe et al. 1993) and analyst activity around the company (D’Mello and Ferris 2000).

As Sect. 2 has shown, the COVID pandemic led to extreme economic conditions with high degrees of uncertainty, which may lead to a more negative announcement effect. However, it is also important to note that the stock market’s condition after the initial shock of the pandemic in March is clearly different from a traditional economic downturn or cold market. Starting in April the stock market appreciated continuously, while high degrees of uncertainty with respect to the pandemic’s development persisted. This period could be described as an uncertain recovery period. Focusing on these high degrees of uncertainty and following Choe et al. (1993) and Bayless and Chaplinsky (1996), we hypothesize:

H1. SEO announcement effects are more negative during the COVID pandemic when compared to non-crisis times.

The second hypothesis pertains to the announcement effects for different industries. Besides looking at SEO announcement effects in the whole market, we subdivide the market according to industry affiliation. According to Myers and Majluf (1984), SEO announcements signal overvaluation. We focus on companies whose business models are positively related to the COVID-19 pandemic.^1^ On the one hand, these “winners” of the pandemic are more likely to be overvalued and therefore experience more negative SEO announcement effects. On the other hand, firms with “the most valuable investment opportunities do not experience adverse stock returns when they issue equity” (Jung et al. 1996). As such, companies with business models that profit from the growth opportunities brought about by the COVID-19 pandemic should experience no or less pronounced adverse stock returns. As these growth opportunities did not just emerge with the SEO announcement but existed before it could be assumed that these should be priced in sufficiently. The new information of an SEO might then signal overvaluation and therefore we hypothesize:

H2. COVID-19 related biotech and healthcare companies experience more negative announcement effects.

## Method and data

### Method

To examine the stock market valuation effect of SEO announcements, we use the event study methodology originally developed by Fama et al. (1969). More specifically, we use the Carhart-4-factor model to model returns, as formulated by Carhart (1997). This model is applied to the estimation period of 100 days, ending 10 days before the respective event date and can be described by the following equation:

$${R}_{i,t}-{R}_{Ft}= {\alpha }_{i}+{\beta }_{i}*({R}_{Mt}-{R}_{Ft})+{s}_{i}*{SMB}_{t}+{h}_{i}*{HML}_{t}+{m}_{i}*{MOM}_{t}+{u}_{it}$$

In order to evaluate the results of the event study we apply parametric and nonparametric significance tests. Firstly, a *t*-test as formulated by Penman (1982) and propagated by Brown and Warner (1985) is applied. Secondly, the adjusted standardized cross-sectional test (later referred to as BMP-test) as developed by Kolari and Pynnönen (2010) is put to use. Besides the parametric tests, we apply the specified Rank test (later referred to as Rank-test) by Corrado and Zivney (1992).

### Data

For the purpose of this study an event is considered if it fulfills the following criteria:

- The SEO was announced between the 11.03.2020 and 30.09.2020.
- The issuing company is based in the United States and listed either on the NYSE or NASDAQ.
- Stock quotations of the issuer’s common stock are available for a period of 110 trading days before the announcement day of the seasoned equity offer.

These restrictions lead to an initial sample size of 512 events which is further reduced by introducing complementary restrictions:

Firstly, to isolate the SEO announcement effect from any confounding event, we conduct a Google news search for the time period around the SEO announcement as well as a review of the investor relations websites of the issuing firms for corresponding press releases. Issues with only minor confounding events until one day before the SEO announcement are kept in the sample due to the (semi-strong) efficient market hypothesis (Fama 1970). While doing this, we also remove rights offerings and private placements.

Secondly, issuers whose stocks are regarded as penny stocks are excluded. Thereby, we follow Bradley et al. (2006) who find that the (semi-strong) market efficiency hypothesis by Fama (1970) does not hold for penny stocks. After the described selection process, the final sample consists of 297 seasoned equity offers made by 278 different issuers.

We further adjust the sample for non-trading times of the NYSE and NASDAQ. The sample firms are obtained from Thomson Reuters by filtering for follow-on offerings. The announcement days are verified through an additional Google news search and an investor relations website review. Stock market data is obtained through Thomson Reuters Datastream. The relevant market data, consisting of the market return, the risk-free interest rate and the Fama and French and the Carhart factors, is obtained from the Data library of Tuck School of Business at Dartmouth College (French 2020).

Table 1 presents the distribution of the 297 sample SEOs along different industries and across time. For this purpose, the 13 industry affiliations provided by Thomson Reuters are consolidated into nine remaining industries. By doing so, the Biotech and the Healthcare industries are recomposed into two industries called Biotech and healthcare as well as COVID-19 related biotech and healthcare. The distinction between these is made on basis of research engagement in the respective field. We define COVID-19 related biotech and healthcare firms as being significantly engaged in developing and providing COVID related test kits, treatments or vaccines. Whether a firm conducts research in the according field is ascertained with a Google search and an assessment of the firm’s investor relations website.

**Table 1:** Offerings per industry and month

Industry	March	April	May	June	July	Aug	Sept	Total	Share
Biotech and healthcare	3	5	26	34	14	19	6	107	36%
COVID-19 rel. biot. and healthc	0	2	6	12	4	8	4	36	12%
Financials	1	2	7	7		3	1	21	7%
High technology	0	2	12	12	4	10	7	47	16%
Real estate	0	0	3	1	2	2	2	10	3%
Media and entertainment	0	0	5	4	1	1	4	15	5%
Retail	0	2	10	8	2	7	2	31	10%
Industrials	1	3	2	7	2	2	2	19	6%
Energy and power	0	1	2	4		3	1	11	4%
Total	5	17	73	89	29	55	29	297	100%
Share	2%	6%	25%	30%	10%	19%	10%	100%	

The Biotech and healthcare and COVID-19 related biotech and healthcare issues make up approximately 48% of the sample. It is important to note that this large share is not anomalous. For the last years the share of SEOs by this industry has been on similar levels, with a 52% share in 2019 (Senior 2020; William Blair 2017, 2018, 2019).

Offerings announced at the transition from spring to summer prevail within the sample, with the highest numbers of offerings in May and June, making up 55% of the sample in total (see Fig. 2). In the following months, the SEO activity declines. Considering the individual industries, the surge in seasoned equity offerings that culminates in June can be observed across all industries.

Table 2 offers several descriptive statistics of characteristics of our final sample.

**Table 2:** Sample description

	N	Mean	Median	25th perc	75th perc
AR_0_ (%)	297	− 8.60	− 6.95	− 14.52	− 1.58
Run-up (%)	297	31.31	23.16	− 0.19	53.16
Lg(Market capitalization) (mio.US$)	297	8.78	8.90	8.09	9.44
Price-to-book	297	8.26	3.21	1.75	7.01
ROE (%)	297	− 42.38	− 20.66	− 72.91	5.42
Relative deal size (%)	297	28.09	14.51	7.68	28.22

This table shows the number of observations (N), mean, median, 25th percentile and the 75th percentile of the variables used in the cross-sectional analysis. The ROE, market capitalization and price-to-book ratio are based on 2019 year-end data. Data is drawn from Worldscope and Thomson Reuters Datastream

## Results of the event study

For the following event study, we focus on the announcement day but also consider abnormal returns from five days before until five days after the announcement in order to provide an assessment of potential early or late reactions of stock prices.

Table 3 presents the results of the event study. Besides the average and median abnormal returns, it contains the test statistics described in Sect. 4. On the announcement day issuers experience a highly significant average abnormal return of − 8.60% and a median abnormal return of − 6.95%. The negative price reaction is highly significant at a 1% level for all relevant tests.

**Table 3:** Abnormal returns upon SEO announcement

Day	AAR	Median AR	*t*-test	BMP-test	Rank-test
− 5	0.64	− 0.12	1.89*	0.90	0.41
− 4	− 0.17	− 0.22	− 0.50	− 0.05	− 0.06
− 3	1.16	0.19	2.34**	2.22***	1.16
− 2	1.05	0.32	2.14**	2.10*	0.48
− 1	0.62	− 0.57	1.15*	1.71**	− 0.46
0	− 8.60	− 6.95	− 11.75***	− 12.29***	− 8.48***
1	− 0.95	− 0.71	− 2.52**	− 2.42*	− 1.13
2	− 0.42	− 0.37	− 1.32	− 1.04	− 0.80
3	0.06	− 0.08	0.24	0.61	0.44
4	− 0.19	− 0.56	− 0.64	− 1.15	− 0.87
5	0.06	− 0.20	0.24	− 0.37	0.14

***, **, * indicate significance at the 1%-, 5%-, and 10%-level, respectively

The consistency in average and median abnormal returns as well as in the test statistics emphasizes that the measured negative price reaction is present in the cross section of the sample and is not disproportionally influenced by few events only. The average and median ARs before and after the announcement day are only weakly significant or insignificant and considerably smaller, indicating an efficient market reaction as well as a well determined announcement date. For robustness purposes we also employed alternative expected return models which have no meaningful impact on the results (see Appendix).

Table 4 shows the CAAR for different event windows. The announcement effect is persistent to changes in the event window’s length. This further supports the findings of Table 3.

**Table 4:** CAAR for different event windows

Event window	CAAR	*t*-test	BMP-test	Rank-test
[− 1,1]	− 8.92	− 9.05***	− 8.05***	− 5.75***
[− 3,3]	− 7.04	− 5.53***	− 5.22***	− 3.32***
[− 5,5]	− 6.02	− 4.60***	− 5.47***	− 2.80***

***, **, *Indicate significance at the 1%-, 5%-, and 10%-level, respectively

The meta study by Veld et al. (2018) reviews 131 studies on SEO announcement effects in the U.S. from 48 influential journals and the SSRN library to consider for the publication bias. The studies find SEO announcement effects ranging from − 7.85 to 3.69%, with an average of − 1.40%. As these studies define different event windows to capture SEO announcement effects, we also group the results with respect to their event window. Each group of event windows contains all event windows lying within the intervals [− 1,1], [0,1] and [0, 5].^2^ The key statistics of the groups are shown in Table 5.

**Table 5:** Key statistics of studies from Veld et al. (2018) for different event windows

	All studies	[− 1,1]	[0,1]	[0,5]
N	131	112	24	26
Mean	− 1.40%	− 1.68%	− 1.35%	− 0.74%
Median	− 1.70%	− 1.79%	− 1.57%	− 0.73%
Max	3.69%	2.48%	1.70%	2.40%
Min	− 7.85%	− 7.85%	− 3.78%	− 3.78%

Our study finds an announcement effect on day 0 of − 8.60% and a CAAR of − 8.92% for the [− 1,1] window. Thus, the empirical findings of this study are more than quintuple the average of the meta study and any of its subgroups.

We therefore conclude that price reactions to SEO announcements are considerably more negative during the COVID-19 pandemic compared to other time periods. This also corresponds with the results of Choe et al. (1993) and Bayless and Chaplinsky (1996), who showed more negative price reactions to SEO announcements during non-expansionary markets. These results are consistent with Hypothesis H1. We can confirm that SEO announcement effects are more negative during the COVID pandemic when compared to non-crisis times.

## Cross-sectional analysis

The cross-sectional analysis aims to examine the potential drivers that influence the heterogeneity of stock price reactions to SEO announcements. To this end, we run a multiple regression with the abnormal stock return on the event day as the dependent variable and the following independent variables:

- Industry affiliations as introduced in Sect. 4, expressed through dummy variables.
- Individual stock run-ups over a period of 60 trading days ending 10 days prior to the event day analogous to Masulis and Korwar (1986).
- Firm valuation as expressed by the price-to-book ratio, similar to Jung et al. (1996).
- Firm size as measured by the logarithm of its market capitalization following Guo and Mech (2000).
- Profitability measured by the return on equity, analogous to Myers and Majluf (1984) or Mueller (1972).
- The deal size of the seasoned equity offering divided by the market capitalization, corresponding to Intintoli and Kahle (2010).

We are primarily interested in whether the SEO announcement effect differs significantly across industries. Latest research provides evidence that government restrictions and social distancing are the main reasons for the strong impact of the COVID-19 pandemic on the stock market (Baker et al. 2020). As certain industries are hit harder by the measures taken by governments, industry affiliation might also explain different reactions to SEO announcements during the pandemic. A particularly interesting industry is COVID-19 related biotech and healthcare, as these companies are affected quite differently by the pandemic in the sense that the pandemic provides earnings growth opportunities for these firms while it is generally bad news for other firms. We also add interaction terms of industry affiliation with the stock run-up and the price-to-book ratio, respectively.

Table 6 shows the estimation results of the cross-sectional regression analysis. Column I thereby shows the regression with the control variables alone which explain 15% of the dependent variable’s variation. The logarithmized market capitalization has a highly significant and positive influence on the announcement effect, implying that larger firms experience less negative abnormal returns upon SEO announcement. This finding is in line with the theory of Guo and Mech (2000) regarding firm size and information asymmetry.

**Table 6:** Cross-sectional regression analysis

	(I)	(II)	(III)	(IV)	(V)	(VI)	(VII)	(VIII)	(IX)	(X)	(XI)
	AR_0_	AR_0_	AR_0_	AR_0_	AR_0_	AR_0_	AR_0_	AR_0_	AR_0_	AR_0_	AR_0_
Constant	− 42.65***	− 45.01***	− 46.05***	− 42.65***	− 45.41***	− 45.66***	− 45.61***	− 45.52***	− 45.82***	− 47.03***	− 46.69***
	(− 6.35)	(− 6.24)	(− 6.59)	(− 6.35)	(− 6.75)	(− 6.82)	(− 6.78)	(− 6.73)	(− 6.80)	(− 6.90)	(− 6.95)
*Controls*											
Run-up	− 0.00	− 0.02	0.00	− 0.00	− 0.00	− 0.00	0.00	− 0.00	0.00	− 0.00	0.00
	(− 0.49)	(− 0.77)	(0.49)	(− 0.49)	(− 0.02)	(− 0.20)	(0.02)	(− 0.03)	(0.35)	(− 0.41)	(0.23)
Price-to-book	0.02	− 0.05	0.03	0.02	0.03	0.03	0.03	0.03	0.04	0.03	0.03
	(1.09)	(− 0.07)	(1.45)	(1.09)	(1.40)	(1.32)	(1.38)	(1.34)	(0.80)	(1.37)	(1.36)
lg(Market cap.)	3.95***	4.19***	4.29***	3.95***	4.21***	4.21***	4.23***	4.23***	4.23***	4.41***	4.36***
	(5.40)	(5.16)	(5.71)	(5.40)	(5.69)	(5.75)	(5.74)	(5.70)	(5.71)	(5.87)	(5.93)
ROE	0.00	0.00	0.00	0.00	0.00	0.00	0.00	0.00	0.00	0.00	0.00
	(1.55)	(1.37)	(1.22)	(1.55)	(1.14)	(1.09)	(1.16)	(1.17)	(1.22)	(0.99)	(1.19)
Rel. deal size	0.02	0.02*	0.02	0.02	0.02	0.02	0.02	0.02	0.02*	0.02*	0.02
	(1.59)	(1.70)	(1.55)	(1.59)	(1.62)	(1.60)	(1.61)	(1.61)	(1.67)	(1.71)	(1.64)
*Industry*											
Biot. and healthc.			0.28								
			(0.17)								
COV-19 biot		− 6.41**		− 6.73**							
		(− 2.13)		(− 2.41)							
Financials		0.19			0.40						
		(0.05)			(0.34)						
High technology		0.29				0.77					
		(0.11)				(0.31)					
Real estate		− 4.99					− 4.75				
		(− 0.27)					(− 0.26)				
Media and entmt		− 0.73						− 0.55			
		(− 0.13)						(− 0.10)			
Retail		0.63							1.02		
		(0.25)							(0.44)		
Industrials		− 0.77								− 0.80	
		(− 0.23)								(− 0.26)	
Energy and power		1.25									1.38
		(0.26)									(0.30)
*Interactions*											
Industry		Yes	− 0.02	0.03	0.04	0.04	− 0.02	− 0.00	− 0.02	0.06	− 0.13
* Run− up			(− 0.94)	(0.85)	(0.34)	(0.73)	(− 0.16)	(− 0.05)	(− 0.83)	(1.43)	(− 1.43)
Industry		Yes	− 0.04	0.76***	− 0.04	0.05	3.46	0.00	− 0.01	− 0.07	0.19
* Price-to-book			(− 0.54)	(2.66)	(− 0.14)	(0.40)	(0.34)	(0.02)	(− 0.26)	(− 0.45)	(0.82)
Observations	297	297	297	297	297	297	297	297	297	297	297
R^2^	0.15	0.18	0.13	0.15	0.13	0.13	0.13	0.13	0.13	0.13	0.13
Adjusted R^2^	0.13	0.09	0.11	0.14	0.10	0.11	0.10	0.10	0.10	0.11	0.11
F-value	6.57***	2.00***	5.42***	6.57***	5.26***	5.56***	5.25***	5.23***	5.33***	5.52***	5.59***

*t*-statistics in parentheses, ***, **, * indicate significance at the 1%-, 5%-, and 10%-level, respectively

Column II includes multiple industry dummies using the biotech and healthcare industry as reference group as well as interaction terms for all industries which are not shown for brevity. With the specified model, the variation in the independent variables explains 18% in the variation of the price reaction on the event day. Columns III to XI test each industry against all other industries and these are our main models of interest. Column IV illustrates that the industry affiliation to COVID-19 related biotech and healthcare as well as the interaction with the Price-to-book ratio are significant at the 5% and 1% level, respectively. COVID related companies experience an on average − 6.73% smaller announcement effect compared to the other industries. This supports Hypothesis H2. Moreover, COVID-19 related biotech and healthcare companies that have a lower price-to-book value experience a more negative announcement effect, which is in line with Dierkens (1991). The model specification in Column IV also provides the highest adjusted coefficient of determination, indicating the best fit of all models.

## Conclusion

Our findings show a quite negative and statistically significant valuation effect of – 8.60% on average for SEO announcements during the COVID pandemic. Compared to prior literature on SEO announcements in non-crisis times this constitutes a considerably more negative price reaction. We show that larger firms have a more positive SEO performance. In the context of the COVID pandemic, one particular explanation might be the reason for this pronounced effect. To limit the negative consequences on a firm level, governments passed bills and enacted instruments to offer additional liquidity. However, as an experience from the global financial crisis, markets are aware that this kind of public support comes fastest and easiest for the largest corporations. This may serve as an explanation for better financing conditions for larger firms. This paper further shows that COVID-19 related biotech and healthcare companies experience a more negative SEO announcement effect of − 6.73% and that within this industry the effect is influenced positively by the firms’ valuation. This presents a remarkable result as this is an exceptional industry in the sense that the pandemic provides earnings growth opportunities for these firms while it is generally bad news for other firms. The fact that these firms exhibit a particularly negative SEO announcement effect points towards lower levels of investor confidence with respect to these firms’ valuation. When being put to test by a SEO announcement, investors adjust their valuation conservatively. Especially COVID-19 related biotech and healthcare firms with a lower valuation experience lower return, as these companies are generally considered to have less earnings growth potential which seems to be further detrimental to the investor confidence. COVID-19 related companies with ongoing growth opportunities and, therefore, only short-term liquidity needs show highly significantly more positive stock market reactions to their announcements compared to firms with lower valuations. All these companies that should profit most from the pandemic nevertheless experience intensified negative market reactions compared to other industries. Nonetheless, investors are highly reflective of the reasons for capital increases in this industry and discriminate between their reaction accordingly.

## Appendix

This Appendix supplies robustness analysis regarding different return models and single industries as driving forces of negative announcement effects.

Additionally to using the Carhart-4-factor model, we calculate abnormal returns upon SEO announcement using the market adjusted model, constant mean return model and the market model. The market adjusted model uses market adjusted returns as applied by Brown and Warner (1985):

$${AR}_{it}={R}_{it}-{R}_{mt}$$

The constant mean return model adjusts the return by the long-term average return of the stock and is applied analogous to Masulis (1980):

$${AR}_{it}={R}_{it}-\frac{1}{{T}_{1}-{T}_{0}}\sum_{t\in [{T}_{0},{T}_{1}]}{R}_{it}$$

Lastly, we use the market model as developed by Sharpe (1964), adjusting for market risk:

$$A{R}_{it}={R}_{it}-({\alpha }_{i}+{\beta }_{i}*{R}_{mt})$$

The following three tables present the results of the event studies using the different return models. They report the AAR, median AR and *t*-test statistics. It can be seen from these results that the expected return model has no meaningful impact on the event day return (Tables 7, 8, 9).

**Table 7:** Abnormal returns upon SEO announcement with market adjusted model

Days	AAR	Median AR	*t*-Test
− 5	0.95	1.10	2.31**
− 4	0.23	− 0.09	0.33
− 3	1.52	0.63	1.67*
− 2	1.11	− 0.69	1.83*
− 1	0.96	− 0.33	1.67*
0	− 8.05	− 7.53	− 7.98***
1	0.14	− 0.03	0.18
2	2.05	0.98	2.64***
3	0.42	0.42	0.40
4	0.83	0.34	0.90
5	2.40	1.42	2.54**

***, **, *Indicate significance at the 1%-, 5%-, and 10%-level, respectively

**Table 8:** Abnormal returns upon SEO announcement with mean return model

Days	AAR	Median AR	*t*-Test
− 5	0.19	1.04	0.45
− 4	− 0.44	− 0.40	− 0.59
− 3	0.57	− 0.44	0.63
− 2	1.07	− 0.85	2.10**
− 1	1.05	− 0.46	2.16**
0	− 8.87	− 8.55	− 9.42***
1	− 0.81	− 0.77	− 1.02
2	1.14	1.14	1.40
3	− 0.30	0.06	− 0.27
4	0.04	0.12	0.04
5	1.74	0.83	1.78*

***, **, *Indicate significance at the 1%-, 5%-, and 10%-level, respectively

**Table 9:** Abnormal returns upon SEO announcement with market model

Days	AAR	Median AR	*t*-Test
− 5	0.69	− 0.12	1.89*
− 4	− 0.19	− 0.22	− 0.50
− 3	1.13	0.19	2.34**
− 2	0.97	0.32	2.14**
− 1	0.66	− 0.57	1.15
0	− 8.63	− 6.95	− 11.75***
1	− 0.90	− 0.71	− 2.52**
2	− 0.40	− 0.37	− 1.32
3	0.11	− 0.08	0.24
4	− 0.23	− 0.56	− 0.64
5	− 0.04	− 0.20	− 0.24

***, **, *Indicate significance at the 1%-, 5%-, and 10%-level, respectively

To further test the robustness of the conducted event study we drop the financial, COVID-19 related biotech and healthcare as well as Biotech and healthcare industries from the sample. We thereby ensure that the negative announcement effect across all observations is not only driven by these single industries. The results of these event studies are reported in the following tables. The initial market reaction on the announcement days is − 8.06%, − 8.20% and − 7.10% when dropping the respective industries. Overall, there are only minor changes to the results on the event day when omitting single industries. Effect sizes as well as significances stay consistent (Tables 10, 11, 12).

**Table 10:** Abnormal returns upon SEO announcement without financial firms with Carhart-4-factor model

Days	AAR	Median AR	*t*-Test	BMP-test	Rank test
− 5	0.95	0.02	2.32**	1.54	0.82
− 4	− 0.31	− 0.59	− 0.81	0.06	− 0.14
− 3	1.34	0.10	2.24**	2.42**	1.12
− 2	1.11	− 0.08	1.84*	1.99**	0.18
− 1	1.03	0.71	1.56	2.11**	− 0.42
0	− 8.06	− 7.56	− 11.52***	− 11.25***	− 8.45***
1	− 1.00	− 0.88	− 2.59***	− 2.63***	− 1.04
2	− 0.54	− 0.47	− 1.60	− 1.14	− 0.74
3	− 0.05	− 0.23	− 0.19	0.47	0.40
4	− 0.17	− 0.68	− 0.49	− 0.82	− 0.70
5	− 0.14	− 0.29	− 0.49	− 0.39	0.12

***, **, *Indicate significance at the 1%-, 5%-, and 10%-level, respectively

**Table 11:** Abnormal returns upon SEO announcement without COVID-19 related biotech and healthcare firms with Carhart-4-factor model

Days	AAR	Median AR	*t*-Test	BMP-Test	Rank-test
− 5	0.70	− 0.12	1.01	1.06	0.45
− 4	0.09	− 0.03	0.26	0.55	0.03
− 3	0.96	0.19	1.86	1.74	1.04
− 2	0.54	− 0.01	1.18	1.14	0.09
− 1	1.05	− 0.37	1.82*	2.36**	0.00
0	− 8.20	− 6.69	− 10.67***	− 11.39***	− 8.45***
1	− 0.68	− 0.59	− 1.69*	− 1.64*	− 0.82
2	− 0.52	− 0.39	− 1.53	− 1.13	− 0.93
3	0.19	0.09	0.73	0.99	0.01
4	− 0.10	− 0.56	− 0.33	− 1.16	− 0.78
5	− 0.13	− 0.26	− 0.53	− 0.87	− 0.04

***, **, *Indicate significance at the 1%-, 5%-, and 10%-level, respectively

**Table 12:** Abnormal returns upon SEO announcement without Biotech & healthcare firms with Carhart-4-factor model

Days	AAR	Median AR	*t*-Test	BMP-Test	Rank-test
− 5	0.50	− 0.22	1.01	0.16	0.00
− 4	0.00	− 0.19	0.00	0.00	0.00
− 3	1.29	0.00	1.93*	1.86*	0.78
− 2	1.17	0.31	1.75*	1.82*	0.69
− 1	− 0.05	− 0.97	− 0.09	0.59	− 0.86
0	− 7.10	− 6.38	− 11.27***	− 12.32***	− 8.46***
1	− 1.07	− 1.21	− 2.46**	− 2.51**	− 1.50
2	− 0.03	− 0.38	− 0.05	− 0.05	− 0.28
3	0.02	0.11	0.30	0.57	0.07
4	− 0.29	− 0.68	− 0.65	− 1.27	− 0.88
5	− 0.15	− 0.25	− 0.43	− 1.54	− 0.10

***, **, *Indicate significance at the 1%-, 5%-, and 10%-level, respectively

## Footnotes

1 The characteristics of these companies are further discussed in Sect. 4.2.

2 E.g., the event windows (−  1,0), (−  1,1), (0,1) lie in the interval [−  1,1].
